# Collectanea Medica, Consisting of Anecdotes, Facts, Extracts, Illustrations, Queries, Suggestions, &c.

**Published:** 1817-07

**Authors:** 


### COLLECTANEA MEDICA,

CONSISTING OF

### ANECDOTES, FACTS, EXTRACTS, ILLUSTRATIONS, QUERIES, SUGGESTIONS, &c.

RELATING TO THE

*History or the Art of Medicine, and the Auxiliary Sciences.*

Quicquid agunt medici, nostri farrago libelli.

### *Transactions of the Medical Society of London.*

*On Epilepsy;* by JOSEPH ADAMS, M.D. Physician to the Small-pox Hospital and to the Central Dispensary, and President of the Society.

[The following paper was made the subject of an anniversary oration before the Medical Society of London so long ago as the year 1806. Its form is somewhat altered; and the lapse of nine years may have corrected the author's mode of expression, but it has served to confirm his opinions.]

THE ancients were more generally attentive than the moderns to one most important division of certain diseases. I mean the difference between their chronic and acute form. The Greek writers, in particular, were careful to mark this distinction in almost every disease; and modern authors, who have acquired celebrity for the accuracy of their descriptions, have not been less attentive to it. The only division which Aretæus thought it necessary to make of diseases in general, was into acute and chronic; and what remains of his labours commences with an unfortunately mutilated account of the acute epilepsy. Sydenham has not been less careful in distinguishing the acute from the chronic gout; and Mr. Hunter has done much to relieve the sufferings of advanced life by the accuracy of his distinctions between the inflammatory and the spasmodic stricture.

Unfortunately, the celebrity of such names has not been sufficient to preserve these distinctions with sufficient accuracy. This is the more to be lamented, because a directly different treatment is often necessary necessary in the chronic and acute form of a disease, known by the same name, and most of all, because the incurable nature of one form has induced some of the ablest men to despair of success in the other. This is so well known of epilepsy and gout, that they have both been called the *opprobria medicorum;* and the frequent return and long continuance of some strictures under every mode of treatment, is, perhaps, only less notorious as the disease is less obtrusive.

In my remarks on epilepsy, I shall, therefore, beg the reader's very particular attention to the two forms under which it appears, because by these it will be proposed to govern our prognosis and regulate our practice.

The acute epilepsy, on the cure of which only I have any thing to offer, is sometimes preceded by higher health than ordinary; at others, for a few days, by heaviness and an incapacity in the patient to arrange the objects of his attention with the usual accuracy. Oftentimes these will cease a few hours before the paroxysm, and be succeeded by an hilarity which, though part of the disease, will, by the friends, be considered as the cause. The first attack is most commonly at night, immediately after or during the first sleep, rendered more inviting and profound by previous exertion or a hearty meal. The patient is altogether insensible during the paroxysm, according to the severity of which will be his subsequent feelings. The duration is not less uncertain; sometimes he will have a temporary respite, during which the terrors of his mind seem beyond all description, till the paroxysm returns with the same violence as before, and with the same insensibility.

The symptoms are universal convulsions, with so preternatural a power in all the muscles, that the strength of several stout men is necessary to restrain the dangerous exertions of even a delicate subject. This muscular action is never uniform, but attended with those alternate contractions and relaxations from which the convulsions arise. The tongue and fauces partake of it so much, that sometimes the former is forced against the teeth with such violence as to bleed by its pressure against the sharp edges; or, if the mouth happens to be open, the tongue will be thrust out before the teeth are closed with such force as to produce a painful division in its substance. The muscles of the abdomen, intestines, and bladder, are so contracted in the most violent form of the disease, as to force out the contents of these viscera; and it is said that the spermatic secretion is sometimes pressed out at the same time. The complexion of the skin, as well as the turgescence of the superficial veins, depends on the respiration of the patient, and, probably, on the irregular contraction of the heart. At one time the whole surface, more particularly the face, will have a dingy purple appearance; and the veins, especially of the neck, as is particularly remarked by Aretæus, will rise much above the skin. As the patient takes a deep respiration these subside, and the complexion recovers. In the mean while, the struggling for breath produces so strong a

4 pressure pressure on the lungs and bronchiæ, that the secretion is, as it were, pumped up, and the patient is said to foam.

The number and force of the symptoms depend on the violence of the paroxysm: the same may be said of its subsequent effects. At first, the patient falls into a profound sleep, or a condition similar to it: he awakes, and appears wild and unconscious of all that has happened. When, however, he attempts to move, he feels a stiffness in all his joints; and, if he speaks, the state of his tongue, in some cases, explains every thing to him. In proportion as he recovers his reason, the impressions on his mind are more painful. Sometimes he continues for a few days deprived of reason, without inclination or necessity for food; at others, nearly in a state of idiotcy painful to the beholder, and apparently attended with most distressing horrors to himself. From these he gradually recovers, and, excepting of the events during the paroxysm, or of those immediately before and after it, his recollection is as perfect as before.

If the cause of the disease continues, however, the respite is only temporary, and the paroxysm returns sometimes with great regularity in its periods, though these may vary in different subjects. The most usual medium is from three to six weeks, but they have varied from a fortnight to a twelvemonth. If the returns are frequent and violent, the patient has not time to recover his reason between them. Each succeeding paroxysm leaves him worse than before, till at length he becomes idiotic or maniacal, or dies apoplectic, during a paroxysm, or suddenly, without any marked symptoms.

Of such a disease, so well-marked and so formidable, there can be no difficulty in ascertaining the true character. If epilepsy is sometimes confounded with hysteria, this can only be in its chronic form, which shall be considered hereafter.

Examination of the head, *post mortem*, never fails to shew the cause of the acute symptoms, by exhibiting the effects of the actions which produced them. If a patient dies of any other complaint, after several acute epileptic attacks, the only difference perceived is in the texture of the brain, which is firmer and more elastic than usual; with the cavities somewhat larger. If he dies from the effect of a paroxysm, a considerable quantity of fluid is found in the ventricles of the brain, which is still firmer, with adhesions of the *pia mater*, and frequently considerable layers of coagulated lymph, more transparent or jelly-like than usual. If he lives sometime after a paroxysm, without perfectly recovering his reason, or with an evident alteration in his temper or character, yet, in other respects, reasonable, we usually find suppuration in the brain, either in different small abscesses, the matter of which has made its way into the cavities and to the basis; or with a whole hemisphere so much altered as, in many places, to render it difficult to distinguish between pus and brain, or with one or more distinct abscesses contained in very thin capsules.

Such is the history of this dreadful disease, which has acquired, in different countries, names according to the impressions it has

 produced produced on the minds of the spectators. In the days of Hippocrates it was imputed, like many others, to supernatural causes. If the work Περι Νοσου Ιερου is really as old as that physician, it must be admitted that he refutes such an opinion by arguments which would not disgrace a much more modern writer. However, the impression of somewhat sacred attached to the disease seems to have been prevalent among the Greeks till their conversion to Christianity. The pythoness, in the Acts, was, probably, an epileptic female, similar to the sybil described by Virgil. Even the Jews, who had the benefit of a purer religion, could not divest themselves of supernatural agency, and, in one instance, the term lunatic* is added to dæmoniac, probably from the periodical returns of the paroxysms. As epileptics recover from their convulsions, and acquire the power of speaking before the full return of their reason, they utter many incoherencies, some of which must be true, and the rest might be interpreted according to the events which followed. Aretæus, however, adopts a name not necessarily connected with supernatural agency, and the etymology of which is too simple to require notice.

Celsus, on this occasion, satisfies himself with the vernacular language of his country, *morbus comitialis vel major nominatur.*

The late Dr. Heberden very justly ascribes the former of these names to the ill omen attached to the disease, and the consequent dissolution of a national assembly, if any person was seized with it.† Among the moderns, the Spaniards and Portuguese, besides the Greek term, have also *gota coral.* A very ingenious Portuguese gentleman assures me, that this is a corruption of *gota incuravel*, or incurable gout; but the Spanish and French Dictionary of Cormon, published at Lyons, derives the word *coral* from the Latin *cor.* The Italians have for their vernacular term *morbo maestro*, or the master disease; the French have a somewhat similar expression, *le mal haut;* and the Germans call it *schwere noth*, or the heavy calamity. All these languages have, besides the above, the medical term *epilepsy*, and in most of them are expressions analogous to our *falling sickness*, (as *mal caduc, malo caduco, fallende sucht.*) This might lead one to suspect, that, in this, as in many other instances, the vulgar have been more correct than the learned, and that they distinguished the chronic disease merely by the sudden *falling*, whilst the acute form was viewed with so much terror as gave rise to expressions they seemed fearful of defining.

The proximate cause of acute epilepsy does not appear to have been sufficiently sought for in the only way in which it could be discovered, by comparing the symptoms with the appearances after

---

* Σεληνιαζομενος, Matt. ch. iv. v. 24, and ch. xvii. v. 15.

† See his learned Commentaries, article Epilepsy. Aulus Gellius speaks of a certain day appointed for soldiers to be sworn in, *nisi* (inter alia) *morbus sonticus* auspiciumve quod sine piaculo preterire non liceat.—N. A. lib. xvi. cap. iv.

death.

death. Hippocrates imputes the whole to a redundance of *pituitâ.* This opinion, venial in his days, seems to have revived in our own times; at least, it is difficult any other way to account for some late remarks on the *pituitary* gland, published on the continent, and transcribed into some of our English journals.

On a review of all the symptoms of acute epilepsy, and of the appearances after death, I have no scruple in referring the whole to inflammation in the substance of the brain. No one is ignorant that in many epileptic subjects no change has been observed in the appearance of that organ; there are, however, many authorities on the contrary side. It does not, therefore, seem unfair to impute this difference to the different forms of a disease, too often included under one general term.

The brain is, if possible, more guarded against injury and spontaneous disease than any other part. Its great importance, and its extreme delicacy, point out the necessity as the cause of these provisions. Against the first, its bony case is admired by every pathologist; against the second, the whole structure and arrangement of its blood-vessels seem particularly directed. The principal means by which the functions of any organ in the living body are impeded, besides external injury, are hæmorrhage and inflammation. Hæmorrhage, in the substance of the brain, seldom fails to produce apoplexy or partial palsy. Inflammation, if continued, destroys the patient by effusion of fluid into its cavities; or by suppuration, the frequent termination of, and a means of relief in, inflammation of other parts; or by action excited and continued beyond what the powers of life can support.

To preserve the brain from hæmorrhage, the veins are not only more numerous and capacious than the arteries, but the sinuses are a most important provision. Hence, whenever the head is too full of blood, either from a plethora or any mechanical obstruction, the capaciousness of the sinuses admits a considerable delay in its return, without injury to the structure of the brain. It would appear, also as if the internal jugular vein, besides its large area, were capable of greater extension than other veins, if Winslow's remark is just, that, "though the largest of all that go to the head, it is not so large as it seems when injected."

By these salutary provisions, the brain may sometimes be loaded with blood for many hours and days with no other inconvenience than a heaviness or drowsiness, of which the patient is very sensible, and the cause of which he often discovers. But, if this fulness is greater than the vessels can support, a rupture of one of them must follow, with the consequences already mentioned. To prevent this, a further provision is made by the process of inflammation. The dangerous effects of inflammation, beyond a certain degree, have been pointed out; it now remains to be shewn in what manner inflammation, when necessary to prevent worse mischief, is conducted, so as to produce its salutary effects without irreparable injury to the parts.

Such is the progress of inflammation in that form which Mr.

 Hunter

Hunter first called the *adhesive* state,—a term now so general, that no apology can be necessary for its adoption. It may not, however, be amiss to say a few words concerning its progress, particularly in the brain. The adhesive, then, is that kind of inflammation in which lymph is thrown out without any rupture of a vessel. This lymph, by its adhesive property, unites, with great rapidity, parts previously only in contact. It produces these effects, according to the structure of parts. When the vessels of the brain are so turgid as to endanger a rupture, the provision made is to strengthen their sides by the coagulated part of the lymph, which, by its *adhesions*, enables them to resist the effects of increased pressure. Hence the brain is firmer and more elastic; an increased quantity of fluid is also found in the cavities, which are readily enlarged according to the capacity required of them. The danger from plethora is thus provided against; and, if not very considerable, the plethora ceases, by the effusion, by the violent actions which follow, and by the generally dejected state of the patient for a few days after; but the brain has undergone the adhesive inflammation, that is, its substance is hardened by the coagulum, and its ventricles are enlarged by the increased quantity of fluid. This lymph and fluid, therefore, must be absorbed before the brain is restored to its healthy state.

Having thus gone through the history of all the symptoms attending an acute epileptic paroxysm, and also those changes which take place in the brain, so as to become what is called the proximate cause of these symptoms, it may not be a loss of time to compare the presumed cause and its effect.

The previous heaviness of the patient is the effect of plethora, which has rendered the vessels of the brain so full as to require the adhesive inflammation to support them. Inflammation is encreased action. It is, therefore, attended with encreased powers, or a greater capacity of bringing original powers into action; hence, the encreased vigour and hilarity which sometimes immediately precedes a paroxysm. The same has been remarked by Sydenham, immediately previous to a gouty paroxysm, and is among the first aphorisms of Hippocrates relative to disease in general. But, when inflammation is very considerable in any organ, the customary functions of that organ are suspended. This is well known in the intestinal canal, and in the kidneys. Even the muscles cease to act under violent inflammation, an effect extended to the muscles of the heart itself. From the cessation of the usual functions of the brain, we should expect a total insensibility to every impression on the organs of sense, and an entire extinction of memory; and such is actually the case as long as the high inflammation or epileptic paroxysm continues. But the muscles are put in action by stimuli and sympathy, without any consciousness of the mind. Hence, the great irregularity of their convulsive contractions, when they are no longer influenced by the mind and their encreased violence, in contracting, probably, from the strong impressions conveyed through the nerves from parts of the brain under the encreased action of inflammation, inflammation. This violent action is, as in all other cases, succeeded by torpor, during which the patient is, or appears asleep. The rigidity of the muscles, for a few days after, is the necessary consequence of their violent exertions, and the dulness of the senses is the effect of the effused lymph. As the lymph is absorbed, the functions of the parts are restored; thus the patient returns to his memory and reason in a greater or less time, in proportion to the violence and length of the paroxysm, and the consequent larger effusion of lymph. Sometimes the quantity of lymph effused is so considerable that the whole is not absorbed before the periodical return of the paroxysm: in this case, a fresh effusion takes place, and the quantity of adhesion in the substance, and of fluid in the ventricles of the brain, is thus gradually accumulated, till all memory is lost and the patient becomes idiotic. If suppuration has taken place, the symptoms subside in part; but the patient is rarely perfectly restored. To the world the difference may not appear, but those immediately about him find his faculties impaired, and oftentimes his temper altered. In this condition the continuance of his life seems to depend on the firmness of the capsules formed by the adhesive inflammation to contain the pus: if these are thin, they suddenly give way, and the patient dies apoplectic, or in an instant without any immediate preceding symptoms.

The above history, it will be remarked, is confined entirely to inflammation excited in the substance of the brain, without external injury. If inflammation attacks the membranes of the brain, the effect is at first intense pain, and afterwards phrenzy. If the brain is injured by mechanical violence, the consequence is coma or convulsion, and sometimes inflammation ending in suppuration, which either destroys the patient or injures the functions of that important organ.

The most common cause of spontaneous inflammation, or inflammation excited without external violence, is plethora, determined to a part from which there is not an easy egress from the body. Plethora is a frequent effect of certain changes produced at particular ages. During growth, a larger quantity of nourishment is necessary, which sometimes produces plethora, when the growth is completed or even during its progress.* In certain parts near the surface, particularly the nose and the rectum, provision is made for relief from plethora, by the increased number of blood-vessels, and the facility with which they are ruptured and heal. If, at these times, the plethora is determined to the head without this relief, epilepsy sometimes follows. As the changes in the constitution are completed, the cause and the effect cease together. Hence, at this age, a paroxysm or two, or more, sometimes occur, after which the patient remains free from any return. These and others occurring

---

* Epilepsy, from this cause, is very common in young dogs: it is sometimes considered as *rabies;* at others the animal is killed from a mistaken wish of putting him out of his supposed misery.

at at a more remote climacteric, are the causes which have given celebrity to certain remedies. The first have also given rise to a very general error, namely, that if epilepsy occurs early in life, it frequently ceases with the change which takes place at puberty. On the contrary, as the late Dr. Heberden remarks, if it commences much before that period, it increases as the vigour of the body increases. Aretæus had before noticed, that, if it continues beyond the vigour of life, it becomes chronic, and, without shortening life, only ceases with the patient's existence. On this account he confines his remedies entirely to the relief of the acute paroxysm, referring the cure of the disease itself, or the prevention of its return, to his remarks on the chronic disease.

With submission to an authority of which I so gladly avail myself in the division of the disease, I shall consider every paroxysm acute which produces any of the consequences above mentioned; but, most of all, temporary loss of memory and its gradual return. These I can only impute to such a change in the condition of the brain as may admit of restoration; that is, to the adhesive inflammation, the progress, termination, and sequel of which has been sufficiently dwelt upon for our present purpose.

[The remainder of this paper will be hereafter noticed in our analysis of the rest of the Transactions.]

---

### *Miscellaneous Observations on Contagious and Infectious Diseases, from* Dr. Gallup, *of America.*

"There was a time, undoubtedly, when contagious and infectious diseases were strangers to this earth; they have had an origin, at different times, by a concurrence of causes unknown to us. It is sufficient for our purpose to learn their present character. Although the appearance of these classes of disease is not precisely the same in all seasons, and in all habits, yet they manifestly have a determined character, and are nearly alike at different times. Those that are assigned to the class of *contagion*, require about the same term of time to run through the system now that they did centuries ago; those of the class of *infection* are governed by the same laws now as in former times.

"It is believed, there is little doubt that most, if not all, the diseases of these classes may be generated, in certain places and seasons, from a recurrence of the causes which first produced them in former times; and that they spread from one person to another, and become epidemic more or less, according to the particular state of the atmosphere, favouring their propagation. But the productions of these kinds are very rare, and their existence not attended with a satisfactory proof which is sufficient to gain the full assent of the mind: the most we know of them is, that they are propagated in a seminal influence; and probably were instituted for the scourge of man.

"With respect to those diseases that we call *epidemic*, every term of experience serves to convince that they are very mutable in their their character. Where a concourse of symptoms designate the disease in popular language, the discerning physician often discovers a difference from those which he has seen on former occasions, called by the same name. This is so manifestly the case, that hardly any two *epidemics* are said to be exactly alike; at any rate, they have not the specific character of the fevers from contagion. This difference is imputed to some variation in the predisponent principle. It is agreeable to the analogy observed in the animal and vegetable kingdoms to suppose, that even the diseases of the most specific character become extinct, and that others are generated from the operations of physical causes. Species of animals did exist in former times, as we learn from authentic history, that have now become extinct, and those of the most perfect kind.

"Certain insects and reptiles are produced, on certain occasions, from a concurrence of circumstances unknown to us, which seem to be of a different character. They are creatures of a day, as it were; some of them real ephemeras, others of longer duration. All of the same species, alike in colour, size, and duration; myriads congregate together, and become extinct together. We have abundant reason to suppose, that these are the production of certain elementary principles: the particular combinations of matter, however, are wholly unknown to us. An analogy is here presented worthy of our attention.

"We are fully apprised of the danger of classing diseases in the ordinary method of nosological arrangement, according to the different types or states of the system that have been assigned to particular diseases. This has been a source of infinite error. It was considered more appropriate to make only some general classifications of fever, founded on their remote causes. If one reasonable argument can be urged in favour of this mode, it will be full as much as can be said in favour of the common nosological arrangements. One important benefit, in a classification by remote causes, will be to draw a discriminating line between those diseases that may have a foreign origin, and those that may have a domestic or elementary origin. The former may be transported from place to place, and may be communicated under all circumstances; whilst the latter are susceptible only in certain places, or under certain circumstances. From the want of this discrimination, great discordance of opinion has prevailed amongst physicians; and much perplexity has arisen in different countries in public and private concerns. From an apprehension that certain diseases of an epidemic character are contagious, the sick have been abandoned to their fate by friends and physicians; the common acts of hospitality, and the necessaries of life, and a common burial, have been denied them!

"Tedious and oppressive quarantines have been established, to the great injury of commerce, and annoyance of individuals. It will not be denied but that quarantines, of short duration, may be necessary, for the purpose of cleaning ships, wearing apparel, &c. of every impurity and fermenting principle, whereby miasmata may be produced; the same as in streets or houses: but forty days' detention for the purification of the bodies of men is useless in epidemic fevers; and the restriction had its origin in a profound ignorance of the laws and principles that govern epidemic diseases.

"The folly of adhering to nosological arrangement is conspicuous in this,—that almost always, when an epidemic is present, the type of disease is different in different cases, and in the same case at different stages: for example, when an epidemic angina prevails, some cases appear more distinctly in the character of what is called synocha, and others more distinctly in that of typhus; and also the states of the system are often so blended between the two extremes, that it is believed the most acute nosologist is put at much strife with himself to determine which genus to place it in. It is divided sometimes, and the mean difference is called *synochus*. Dr. Cullen, with all his nosological ingenuity, would find no place for putrid fever, notwithstanding it was so common, and often appeared in intermittent fever, &c. His other works stand, perhaps, unrivalled. It may be added, that nosology would be at least innocent, if it did not lead the student to prescribe for names, and neglect the degree of morbid excitement and real character of the disease.

"The disease should be treated according to what it is, and not according to any generic character it may have forced upon it. The spotted fever seems yet unaccustomed to the yoke of nosological restraint, notwithstanding the attempts to subdue it. Perhaps there is no disease in history that is so variable in its phenomena, and requires such versatility of treatment. It is a conspicuous example of the variety of febrile action, and is well calculated to test the skill of the physician.

---

